# Animal Welfare Assessment: Can We Develop a Practical, Time-Limited Assessment Protocol for Pasture-Based Dairy Cows in New Zealand?

**DOI:** 10.3390/ani10101918

**Published:** 2020-10-19

**Authors:** Sujan Sapkota, Richard Laven, Kristina Müller, Nikki Kells

**Affiliations:** School of Veterinary Science, Massey University, Palmerston North 4442, New Zealand; R.Laven@massey.ac.nz (R.L.); K.Mueller@massey.ac.nz (K.M.); N.J.Kells@massey.ac.nz (N.K.)

**Keywords:** welfare assessment, pasture-based dairy cows, New Zealand

## Abstract

**Simple Summary:**

Systematic welfare assessment protocols are increasingly being used as a tool to demonstrate animal welfare and to drive improvements within the industry. Despite dairy products from New Zealand trading on the ‘green’ image of extensive pasture-based farms, dairy cattle welfare is not routinely assessed on most New Zealand farms, and there is no industry-recognised protocol for such assessment. Drawing on protocols and studies from across the world, this project aimed to create a science-based but practical assessment of dairy cow welfare that could be undertaken as a single one-day visit with a focus on assessment around milking time. After in-farm testing, this project identified 32 assessments which could form a part of such a protocol. Although further testing is required, this protocol could form the basis of a standardised assessment of dairy cow welfare on New Zealand dairy farms.

**Abstract:**

Despite being a leading producer and exporter of dairy products, New Zealand has no industry-recognised welfare assessment protocol. A New Zealand-specific protocol is essential, as almost all dairy farms in New Zealand are pasture-based and housing is rarely used. Therefore, protocols developed for intensive cows are not suitable. The aim of this study was to develop a simple yet practical welfare assessment protocol that could be used to assess the welfare of a dairy herd during one visit timed to occur around milking. Six welfare assessment protocols and four studies of dairy cattle welfare assessments that had some focus on dairy cattle welfare at pasture were used, along with the New Zealand Dairy Cattle Code of Welfare, to identify potential assessments for inclusion in the protocol. Eighty-four potential assessments (20 record-based and 64 that needed assessing on-farm) were identified by this process of welfare assessments. After screening to exclude on-farm assessments that were not relevant, that had only limited practical application in pasture-based dairy cows or that required more time than available, 28 on-farm assessments remained, which were put together with the 20 record-based assessments and were tested for feasibility, practicality and time on two pasture-based dairy farms. Assessments were then identified as suitable, suitable after modification or not feasible. Suitable and modified assessments were then included in the final protocol alongside additional measures specific to New Zealand dairy farms. The final protocol included 24 on-farm assessments and eight record-based assessments. Further testing of these 32 assessments is needed on more dairy farms across New Zealand before the protocol can be used to routinely assess the welfare of dairy cows in New Zealand.

## 1. Introduction

Social demand for quality animal products from welfare-friendly farms has led to the development of a myriad of welfare assurance schemes which set a higher benchmark for animal welfare than legislation, especially in Europe [1]. However, there is a growing need for robust and science-based welfare assessment systems all over the globe. Despite being the eighth largest producer of milk [2] and a leading nation in terms of milk exports [3], New Zealand still has no industry-standard welfare assessment scheme and routine welfare assessments are rare on New Zealand dairy farms. In contrast, the Red Tractor scheme, which requires regular independent assessment of animal welfare, certifies 95% of milk produced in the UK [4]. Having such a scheme could be a significant boon for the New Zealand dairy industry, as it would provide transparency regarding the general welfare status of cattle on New Zealand dairy farms, as well as benchmarking to drive continued improvements in animal welfare [5].

Starting with the development and codification of the Five Freedoms [6], through to the conceptualisation of the Five Domains model, a strong science-based foundation has been established for the development of systematic, structured, inclusive and logically consistent welfare assessments protocols [7]. In the Five Domains model, the first four physical/functional domains (nutrition, environment, health and behaviour) are concerned with biological functioning/physical wellbeing and the fifth domain, the mental state, is concerned with affective state or psychological wellbeing [8].

Most welfare assessment protocols amalgamate animal, resource and management-based assessments [9], along with assessments related to stockpersonship and farm records [10]. Much of the emphasis in such protocols is on outcomes (i.e., animal-based assessments) rather than inputs (resource- and management-based assessments), as animal-based assessments reflect the actual response of the animals to the inputs, rather than the potential response to those environments and management practices [8]. Nevertheless, input-based measures are still commonly used as part of farm-animal welfare assessment protocols because of the ease with which they can be measured and benchmarked [11]. They are also particularly useful as predictors of potential welfare issues when the welfare assessment protocol is being undertaken as part of a single visit rather than an ongoing repeated observation.

Welfare assessment protocols need to be relevant to the management system in which the animal is being kept [12]. This means that before protocols designed for welfare assessment in one system are used for assessment in a different farming system, they must be tested for their relevance, as protocols suitable for one system may not be suitable for a different system. For example, Laven and Fabian [13] studied the practicalities of using a welfare assessment designed for housed dairy cows [9] on dairy farms in New Zealand where cattle were permanently kept at pasture and concluded that significant refinements to the protocol were needed before it could be used on New Zealand dairy farms. However, the focus of that study was on determining lameness prevalence on those dairy farms, rather than welfare assessment, and the welfare assessment was limited to animal-based assessments that could be evaluated alongside whole herd locomotion scoring. No assessments of resources, animal handling or stockpersonship were included in their study.

If an assessment protocol is to be used widely, it needs to include individual assessments that are practical to measure within the system being assessed, and it needs to be achievable within a reasonable time frame [14]. The latter is particularly important in a pasture-based system, as many animal-based assessments are only measurable while cattle are being milked, as this is the only time that they can be closely and systematically observed. As time during milking is limited, this means that even to collect data from a relatively small number of cows, animal-based assessments will require either two observers or observations during at least two milkings.

Thus, the aim of this study was to expand the assessment made by Laven and Fabian [13] and develop a welfare assessment protocol for dairy cows (based on a combination of animal-based, resource-based and stockpersonship-based assessments) that would be suitable for farms adopting the pasture-based dairy farming system which predominates in New Zealand. In addition, the protocol needed to be achievable within a single day assessment period, in which one person collected data before milking (for a maximum of 2 h) and two people collected data during afternoon milking.

## 2. Materials and Methods

**Figure 1:**
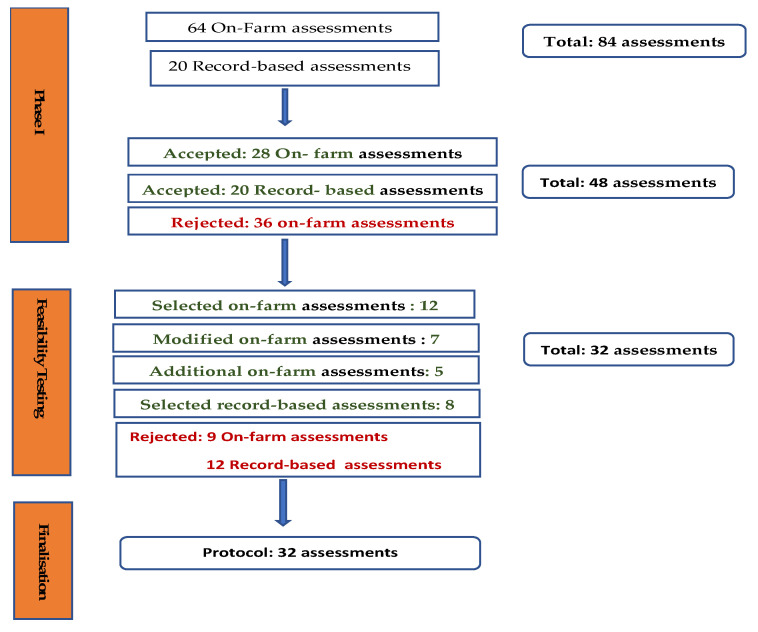
Summary of identification, screening, and finalisation of welfare assessments included in the protocol for assessment of welfare in pasture-based dairy cows in New Zealand.

The research was undertaken in three phases (Figure 1).

### 2.1. Collection and Screening of Potential Assessments

The base protocol used was the Welfare Quality protocol for dairy cows [13]. As this was not specifically designed for cattle kept permanently at pasture, additional potential assessments were identified from five assessment protocols which had some focus on cattle at pasture. This was a convenience-driven selection, designed to reflect welfare assessments used across the world, with two from Europe (AssureWel [15] and the Animal Need Index [16]); two from North America (the UC Davis Cow-Calf Health and Handling Assessment [17] and the Canadian Animal care module [18]) and one from South America (the Chilean Protocol of Animal Welfare for Dairy Farms [19]). This was then supplemented by data from four studies on dairy cattle welfare assessment—three from Europe [9,20,21] and one from South America [22].

The welfare assessments identified by this process (see Appendix A) were then screened by the authors to identify and exclude (a) assessments that were only relevant to non-pasture-based dairy farming systems,(b) assessments that had limited practical application in such a system, or (c) assessments that required significantly more time than would be available in a one-off single day assessment.

The remaining assessments were then used to create a protocol for feasibility testing. Prior to this testing the protocol was checked against the New Zealand Dairy Cattle Code of Welfare, to confirm that no potentially relevant areas of welfare covered by the code had been omitted.

### 2.2. Feasibility Testing

Feasibility testing of the test protocol was undertaken on two dairy farms in the Manawatu region of the North Island of New Zealand—one where cattle were milked on a rotary platform (Farm 1) and one where cows were milked through a herringbone (Farm 2).

The first feasibility test was undertaken in June 2019 on Farm 1 during the milking of 198 autumn-calving cows.

All resource-based assessments were tested before afternoon milking, whereas animal-based and stock skills-related assessments were assessed during the afternoon milking (see Appendix A and Appendix B for selected assessments considered for feasibility testing). All assessments were evaluated for practicability, time taken, best place for assessment and ease of scoring. For animal-based assessments the intention was to score/assess all animals. In addition, the best site for recording those assessments was evaluated by two assessors; one who stood outside the parlour (in a good site for locomotion scoring) and one inside the parlour. The assessor outside the parlour attempted to record rumen fill, body condition, skin injuries, broken tail, cleanliness, coughing, nasal and ocular discharge and diarrhoea, alongside locomotion score, whereas the assessor inside the parlour attempted to record the same assessments without also recording the locomotion score.

Record-based assessments were collected after milking using a questionnaire (Appendix B) combined with a farmer interview if the farmer had time available. At the end of this first test the evaluated assessments were divided into three groups based on the consensus of the four authors: (i) assessments that were not suitable for inclusion as part of a one-off single day assessment; (ii) assessments which were suitable, but which needed modification (e.g., changes in the scoring system) and (iii) assessments which were identified as suitable for inclusion in the final protocol without change. Alongside this categorisation, additional assessments were identified of which the inclusion would increase the comprehensiveness of the assessment protocol. Finally, for all the suitable animal-based assessments, a conclusion was made as to whether they were best assessed while the cows were being milked or outside the milking parlour.

This revised assessment protocol was then tested again on Farm 1 in July 2019 (excluding the resource-based and record-based assessments, as their practicability had already been tested on this farm). At that time, 900 spring- and autumn-calving cows were being milked. The final trial of the protocol was undertaken on Farm 2 (milking ~300 cows; all assessments evaluated) in October 2019.

### 2.3. Finalisation Phase

After all three farm visits had been completed, a critical assessment was made of the assessments included in the protocol used for farm visits 2 and 3 and the final protocol was confirmed.

## 3. Results

During phase I, 84 potential assessments were identified (see Appendix A and Appendix B). Of these, 20 were assessments based on farmer records/recollection (see Appendix B) and were therefore to be collected using a questionnaire. This meant that there were 64 remaining assessments which would require active on-farm measurement, and therefore required screening during the first phase. Of these 64, 21 were excluded because they were irrelevant under New Zealand conditions, 13 because they had limited practical application under New Zealand conditions, and two because it was clear that they would be too time consuming to undertake alongside the other suggested measurements (see Appendix A). This meant that 48 assessments (28 of which required active on-farm measurement (see Appendix C for details of these assessments) and 20 of which were to be answered by questionnaire) were brought forward for feasibility testing (Figure 1).

During phase II (feasibility testing) 21 of these 48 assessments were identified as not being suitable for inclusion as part of a one-off single day assessment protocol where animal-based assessments were principally made during milking. The excluded assessments and the reasons for their lack of suitability are shown in Table 1.

In addition, seven assessments were modified and scores for animal-based assessment were dichotomised (Table 2). Five new assessments were added (Table 3). Of these five assessments, four were related to the handling of cattle before they were milked and are associated with cow flow and lameness risk [23], whereas the fifth, heifer mortality, was added to get better data on mortality before first calving. This left 32 assessments, which were taken to the final two farm trials (Figure 1). The final two farm trials confirmed that the 32 assessments were all suitable and practicable. The final protocol is summarised in Table 3.

## 4. Discussion

The aim of this study was to develop a protocol that could be used to assess welfare on pasture-based dairy farms in New Zealand, where the amount of time available was limited to one person collecting data for up to 2 h before milking and two people collecting during afternoon milking.

The assessments initially included in the protocol were primarily based on the Welfare Quality protocol [11] with changes to reflect the pasture-based system which predominates on New Zealand dairy farms. Assessment protocols that adapt the Welfare Quality protocol to different systems often just use that protocol with some assessments removed and assessments added from another protocol (e.g., [22,28]). In our first phase, we used the same approach but added assessments from a convenience selection of protocols and studies of protocols from across the world, rather than relying on a single additional protocol. This process was not intended to identify all potential assessments but to identify a large range of assessments that could be practically assessed in a test protocol under New Zealand conditions.

Our initial test protocol had 48 assessments (20 record-based and 28 which required active on-farm assessment). Of those 48 assessments, 21 (12 record-based and 9 on-farm) were excluded during the farm trials. As five new assessments were identified during phase 2 (farm trial), the final protocol included 32 assessments, of which 8 were record-based and 24 required active on-farm measurement.

This protocol met the requirement of taking less than two hours to undertake the assessments to be done outside of milking time (paddock or track; see Table 3) and having milking time assessments that could be undertaken by a maximum of two people. However, these time limitations do mean that not all potentially useful assessments were included in the final protocol, which could have compromised the coverage of the assessment protocol.

Mellor (2017) [7] identified four functional domains (“Nutrition”, “Environment”, “Health” and “Behaviour”) as representing the key general foci of animal welfare management. These four domains all need to have sufficient depth and breadth of assessment if a welfare assessment protocol is to properly measure animal welfare status. It is thus critical to assess whether our final protocol meets these requirements.

### 4.1. Nutrition

In this domain, body condition score (BCS) and rumen fill were the two assessments related to feed intake identified by the literature search that were kept after screening and feasibility testing. BCS is an effective measure of energy balance over the medium term [29,30], whereas rumen fill is a reliable measure of feed intake in the previous 24 h [31].

Generally, extreme BCS (too low or too high) is associated with compromised welfare, but there are no clear or simple thresholds for BCS which determine that welfare has been compromised [29]. For this study, we only used a single threshold for low BCS, because very high BCS ≥7 (1–10 score) [32] is very rare on New Zealand dairy farms [13]. The threshold we used for low BCS (≤3) is the same as used previously [13] and is derived from the Dairy Cattle Code of Welfare [33] which states that when BCS is <3 urgent remedial action must be taken. This threshold is well below the optimum BCS for productivity [29].

Rumen fill has been shown to accurately reflect feed intake in the previous 24 h [31]. For this study, a score of ≤2 was determined as indicating poor welfare [26]. The main issue with rumen fill as a measure of nutrition-related welfare is that current health status can also affect it. However, when recorded at the herd level, poor rumen fill is much more likely to reflect feed availability, rather than individual cow health.

Nevertheless, although both BCS and rumen fill accurately reflect medium term energy balance and recent feed intake, respectively, their use in a single one-off assessment may not accurately reflect true welfare status within the nutrition domain. This is because the dependence on grazed grass of New Zealand dairy farms means the balance between feed supply (from pasture) and feed demand in New Zealand is highly seasonal [24]. Multiple measurements of BCS and rumen fill throughout the year may thus be a useful addition to this protocol to take account of seasonal variation in feed supply. This may be particularly important in winter, when negative welfare related to feeding is most likely to occur, especially in dry cows where pasture intake may be restricted to minimise the impact of winter grazing on grass growth in the spring. This negative welfare is not likely to be reflected in BCS, as this will be at its nadir in early lactation rather than during the dry period [34], but it may be reflected in rumen fill if feed restriction is too great.

Assessment of water quality and availability is a crucial aspect of any welfare assessment scheme. For this protocol, average distance between water troughs within a paddock was chosen as the measure of water availability, whereas the cleanliness of water troughs was identified as a suitable measure of water quality. The latter is consistent with the Welfare Quality assessment protocol [11], in which the cleanliness of the water trough is the only measure of water quality. In contrast, water availability in the Welfare Quality assessment protocol includes more assessments, including water flow and size of drinking troughs. These were excluded during the screening process for this study on the basis of the time required. Measuring water flow would require measuring flow in multiple troughs across a farm (to account for distance from central supply); thus, it was excluded for being too time consuming. The distance between drinking troughs was chosen rather than the size of drinking troughs because it was thought to be more applicable to extensive systems [35] where cattle drinking behaviour is different from that of confined cattle [36]. Further research is required on drinking behaviour in cattle at pasture so that the impact of differing types of water provision on cattle welfare (both positive and negative) can be determined.

### 4.2. Environment

The final protocol had twelve assessments in this domain. The majority of assessments (seven) related to cow handling before and during milking. This is a crucial time for the welfare of the pasture-based cow as most welfare issues are likely to arise during this period. Of the five added assessments, four were in this domain. All four were related to cow handling and are crucial assessments for identifying problems with cow flow (i.e., how well the herd moves into the collecting yard and through the milking parlour). Cow flow is crucial on pasture-based farms because it has significant effects on milking time, staff patience and cow welfare (both behavioural and health, especially lameness) [23].

Another measure included in the final protocol that was related to cow flow was the maximum waiting time before entering the milking parlour. This identifies the length of time spent standing on concrete and away from pasture. For small herds (e.g., 200 cows) this can be identified by simply recording the start and end of milking. However, on farms with more cows, cows are generally separated into multiple herds and these are all milked through the same parlour. In such herds (e.g., Farm 1 when it was examined the second time) it was not possible for the assessor inside the parlour to identify when the first herd finished and the next herd arrived. Thus, recording the maximum time waiting should be undertaken by the assessor recording the locomotion score.

Noise level during milking is also associated with cow flow, but is also a measure of the impact of the environment on the cow during milking, as excessive noise can have an adverse effect on an animal’s physiological, behavioural and production aspects [37]. This may be especially important in pasture-based dairy cows as they generally only encounter loud background noises when they are being milked. In dairy cattle, research suggests that noise as high as 80 dB seems to have no effect on milk production [37]. As normal conversation is around 60 dB [38], using a simple categorical measure of noise based on the ease of having a conversation is able to identify when there is no risk of noise-related welfare effects (conversation easily heard) and a high risk (normal conversation not possible) without expensive equipment. However, we recommend that further research is carried out to better establish the effect of noise during milking, specifically in cows based at pasture.

Tracks are a critical part of a pasture-based farm’s infrastructure. In this protocol we included assessments of track quality, length, width and camber, in addition to handling on the track, all of which have been associated with lameness in New Zealand dairy cattle [39]. Track assessments were limited to tracks within 100 m of the milking parlour. These areas are the most used parts of the track on a farm and are thus the most likely to have an impact on lameness risk if they do not meet recommendations. Assessing all the tracks was not feasible within the timescale but would have added little to the 100 m selection and complicated welfare assessment (e.g., is a very poor quality track used once a month equivalent to a good section used daily?).

Two assessments were included which evaluated the environment in which the cows spend most of their time, i.e., the pasture. The cleanliness of the cow reflects the status of the pasture as a clean dry surface to stand and lie on. Compared to the assessment in the Welfare Quality protocol [11] (for housed cattle), there were two key modifications in this assessment. Firstly, measurement of cleanliness of the lower leg (below the hock) was not undertaken (as this not a good reflection of the acceptability of a pasture environment), and secondly, only dried dirt (which reflects persistent rather than one-off contamination) was considered. Both of these modifications were also recommended by the UC Davis Cow-calf handling assessment [17].

As cattle at pasture have an uncontrolled environment, exposure to extreme climatic conditions has been one of the welfare concerns in pasture-based dairy systems [40]. Heat and cold stress can be identified by behaviour, but as the risk of such stress on any individual day is low [41], identifying these behaviours is of very little value in a routine welfare assessment. The best assessment is thus a resource-based one which identifies the resources available should there be a problem. Measurement and assessment of shelter belts and shade provides such a resource-based assessment, but it is time consuming to do this. The major issue is that the availability of shelter does not necessarily indicate that it is used when needed. This information may be possible to collect via a questionnaire, but for such a question there is a clear risk of the owner giving the answer that they think the questioner wants to hear.

### 4.3. Health

Nine animal-based health assessments were included in the feasibility testing, with six being included in the final protocol. Three assessments were excluded because they were not recordable during the examination (nasal and ocular discharge) or because normal could not be separated from abnormal (diarrhoea). Of the remaining six assessments, it was decided that two (blind eye and ingrown horn) would not be specifically counted, but would be recorded if observed (similar to the way that broken tails are assessed in many other assessment protocols [42]). The number of animal-based assessments in the final protocol is much lower than that used in other protocols and studies (e.g., [9,11]). Although some of this is related to limited time (and the requirement to examine cows during milking) (e.g., claw confirmation), the difference is mainly due to many animal-based health assessments used in previous protocols being irrelevant in pasture-based cattle (e.g., swollen and ulcerated hocks).

No sampling procedure was used in this protocol. The aim was to assess all cattle being milked. This is partly because no sampling protocols have been validated for use in pasture-based cattle, but also, in contrast to assessment in housed cattle where sampling significantly reduces the time taken for the assessment, when milking cows are being assessed the assessor still has to be present for the whole of milking, so no time is saved [41].

### 4.4. Behaviour

Seven assessments in this domain were considered during feasibility testing. Four of these were assessments of cattle in the paddock (identifying (i) social agonistic behaviours, (ii) qualitative behaviours, (iii) positive behaviours and (iv) aversion distance) and the remainder were assessments made during milking.

It was in this domain that the time limitations had the most impact on our ability to assess welfare. The Welfare Quality assessment protocol [11] includes a large number of behavioural assessments which take a considerable amount of time to assess. In this protocol there was about 30 min available to assess cow behaviour in the paddock. This limited the assessment to only two of the four in-paddock assessments: social agonistic behaviour and positive behaviours. Qualitative behaviour assessment was excluded because it takes considerably more time than assessing social agonistic and positive behaviours; for example, the Welfare Quality assessment [11] allocates 150 min to assessing this type of behaviour in housed cows. Measurement of aversion distance could not be undertaken alongside behavioural assessment. It was therefore modified and simplified so that it could be assessed during milking (fear, neutral or curious response to the assessor moving towards them at the entrance to the milking parlour).

The available time for in-paddock assessment could have been increased by having the second assessor start at the same time as the first one. However, as milking generally takes around two hours, having both assessors available from two hours before milking would mean that this protocol would take eight hours of assessor time (two times four hours), which is more time than the Welfare Quality assessment generally takes in housed cows [11]. In addition, it is unclear whether the increased time (which is still limited to <2 h) would significantly advance our understanding of welfare within the behaviour domain [25] and how much influence behaviour in the paddock has on a dairy cow’s welfare status.

One key measure of cow behaviour which we were unable to include in the final protocol was cow behaviour during milking. This reflects cow welfare in terms of their mental state in relation to coming into the milking parlour, as well as whether there is current discomfort due to being milked. This did not prove possible to collect alongside other assessments during milking, especially in a rotary parlour. Cow behaviour during milking is related to cow flow (as cows that are not reluctant to be milked will enter the parlour without having to be coerced) and so measuring the response to the assessor and assessing cow handling during milking is likely to provide some of the information which records behaviour during milking.

## 5. Conclusions

The aim of this study was to develop a practical and feasible but science-based welfare assessment protocol for a one-off single day assessment for pasture-based dairy cows in New Zealand. We believe that within these constraints, we have succeeded in creating a protocol with good coverage of the four domains outlined by Mellor which will identify the key areas of welfare concern on a farm, which will be useful in benchmarking and in providing transparency in regard to the welfare of dairy cows on New Zealand farms. However, before this protocol can be used as a basis for welfare assessment on New Zealand dairy farms it needs further testing on more farms in multiple areas of New Zealand.

## Figures and Tables

**Table 1:** Dairy cow welfare assessments that were trialled on-farm but excluded from the final welfare assessment protocol and the rationale for their exclusion.

Rejected Assessment	Reason for Rejection	Rationale
Nasal discharge Ocular discharge	Difficult to observe	During milking (in both types of parlour) the assessor is behind the cows and cannot easily observe the nostrils or the eyes. Outside the parlour, observer needs to be distant from the cows (to avoid interfering with movement), so assessment of mild-moderate discharge was difficult.
Diarrhoea	Difficult to identify pathological diarrhoea	Lactating dairy cattle in New Zealand are fed a low dry matter, highly digestible diet [24], thus loose faecal consistency is normal.
Heat stress indicators Cold stress indicators	Occurrence depends on environmental conditions	Observing such symptoms on a one-off visit is unpredictable. Resource-based assessments, such as assessment shelter, will provide an estimate of the resources available to the cows should there be heat or cold stress.
Slope before the entry and exit of the parlour	Limited resources	Difficult to assess without specialised equipment. Limited impact where track quality is maintained.
Milking behaviour	Difficult to observe alongside other measurements	On an elevated platform in a rotary parlour, only a few cows are observable at any one time. In a herringbone parlour, it was difficult to assess alongside other assessments requiring scoring of individual cows.
Orientation of the cows in the milking yard (facing or back to the parlour)	Not suitable for all NZ farms	Suitable for rectangular yards only.
Qualitative behaviour	Difficult to assess during milking and insufficient time available during paddock visit	Assessment of qualitative behaviours is difficult and time consuming. In paddock Assessment was limited to agonistic and positive behaviours, which were thought to be more important [25].
Disease records	Record quality generally poor—farmer recollection rather than records	Only clinical mastitis and lameness cases were retained. For data collected by outside bodies (e.g., bulk milk somatic cell count), it was thought best to collect from those sources rather than from the farmer.

**Table 2:** Welfare assessment criteria that were modified before inclusion in the final protocol and rationale for their modification.

Assessment	Changes Made	Reason for Modification
**Scoring system (animal-based assessments)**	Only welfare-compromised animals (e.g., score ≥2 for locomotion score, ≤2 for rumen fill and ≤3 for body condition score) recorded.	Categorisation made these assessments simpler and quicker.
**Cleanliness of the cow**	Three-category AHDB cleanliness scoring system [26] used rather than four-category Wisconsin Hygiene Scoring system [27]. Dirtiness on lower leg (below hock joint) and only dried dirt was recorded (fresh dirt was not included in score [17]). Udder, flank and upper leg scored separately. Proportion of cows with at least one score ≥ 1 recorded.	AHDB scoring system and categorisation made assessment simpler and quicker.
**Fear behaviour**	Response of the cows towards the assessor, i.e., fearful, neutral and approach, was observed at the entrance to the parlour. Approximately 2% of cows were assessed.	Interpretation of aversion distance is uncertain in extensive systems [21]; in addition, measurement was not possible in the paddock alongside behavioural observation due to time restrictions.
**Body condition score (BCS)/Skin injury**	Change of site in herringbone parlour from inside to on immediate exit. No change for rotary, as all cows were assessable.	For BCS, only 50% of cows (left row) were assessable in the herringbone parlour. For skin injury, assessment was limited to the back of the cow and one observable side only.
**Maximum waiting time before entering the milking parlour**	Assessor standing outside the parlour recorded arrival times of cow groups/herds.	On farms with multiple herds, the assessor standing inside the parlour could not observe the arrival time of the second herd alongside other assessments.
**Ingrown horn/Blind eye**	Not included under main assessment but recorded if seen.	Difficult to observe systematically but needs to be recorded if observed.

**Table 3:** Final proposed assessment protocol for assessing welfare of pasture-based dairy cattle on New Zealand farms with respective site of assessment.

Welfare Domain [4]	Assessments	Assessment Types	Site of Assessment			
			Inside Parlour	Outside/Around Parlour/Collecting Yard	Paddock or Track	Questionnaire
**Nutrition**	Body condition score ^†^	Animal-based	✔			
	Rumen fill Score	Animal-based	✔			
	Distance to water points	Resource-based			✔	
	Trough cleanliness	Resource-based			✔	
**Environment**	Cow Cleanliness ^†^	Animal-based	✔			
	Shelter availability	Resource-based			✔	
	Maximum waiting time in the collecting yard ^†^	Resource-based		✔		
	Noise level	Resource -based	✔			
	Mixing of Cows	Management-based				✔
	Handling aids	Management-based				✔
	Handling during milking	Stockmanship-based	✔			
	Farthest paddock distance	Resource-based				✔
	Track condition	Resource-based			✔	
	Head position *	Animal-based		✔		
	Handling on track *	Stockmanship-based			✔	
	Yard space per cow *	Resource-based		✔		
	Backing gate speed *	Resource-based		✔		
**Health**	Pain relief	Record-based				✔
	Lameness	Animal-based		✔		
	Broken tail	Animal-based	✔			
	Coughing	Animal-based	✔			
	Skin Injury ^†^	Animal-based	✔			
	Ingrown Horn ^†^	Animal-based		✔		
	Blind eye ^†^	Animal-based		✔		
	Vaccination record	Record based				✔
	Lameness per year	Record-based				✔
	Mastitis per year	Record-based				✔
	Cow mortality per year	Record-based				✔
	Replacement heifer deaths before calving/year *	Record-based				✔
**Behaviour**	Agonistic behaviour	Animal-based			✔	
	Positive behaviour	Animal-based			✔	
	Fear behavior ^†^	Animal-based	✔			

* indicates assessments added to the final protocol during the trials (details on method of assessment in Appendix C) ^†^ indicates assessments which were modified before inclusion in the final protocol (see Table 2 for how they were modified). No superscript indicates assessments that remained unchanged from the initial test protocol to the final protocol.

## Appendix A

**Table A1:** Welfare Assessments Identified during Phase I and Status after Screening.

Welfare Domain [4]	Measuring Standards Taken from Different Protocols Studies and Welfare Code	Status after Screening
**Nutrition**	**Body condition score **	**Accepted**
	**Rumen fill score **	**Accepted **
	**Cleanliness of water points/troughs.**	**Accepted **
	**Average distance to water points from the pasture**	**Accepted **
	**Additional feeding sites in the pasture**	**Limited practical application**
	**Contamination of the feeding site**	**Limited practical application**
	**Feeding places per cow**	**Limited practical application**
	**Distance from the pasture to the feeding site**	**Not relevant at pasture**
	**Choice of water temperature**	**Not relevant at pasture**
	**Proper flow and functioning of the water points**	** Time consuming as part of assessment**
	**Sufficient amount and size of drinking troughs**	** Time consuming as part of assessment**
**Environment** **Health**	**Noise level (e.g., dogs/machineries)**	**Accepted**
	**Cleanliness of udder, lower hind legs (including the hock), hind quarters-upper hind leg, flank and rear-view including tail **	**Accepted**
	**Track condition: surface, width, slope**	**Accepted**
	**Shelter: Provision of shade, wind breaks and natural barriers during extreme weather.**	**Accepted**
	**Heat stress indicators (seeking shade, open mouth panting).**	**Accepted**
	**Cold stress indicators (shivering, huddling, facing away from wind or rain).**	**Accepted**
	**Slope before the entry and exit of the parlour**	**Accepted**
	**Maximum time waiting before entering the milking parlour**	**Accepted**
	**Slope of pasture**	**Limited practical application**
	**Milking hours aligned to climate**	**Limited practical application**
	**Access to the pasture (days per year, average time spent on pasture per day).**	**Limited practical application**
	**Time taken to lie down**	**Not relevant at pasture**
	**Animals colliding with housing equipment while lying down**	**Not relevant at pasture**
	**Animals lying partly or completely outside the lying area**	**Not relevant at pasture**
	**Comfortable calving pen, separate pens for calves**	**Not relevant at pasture**
	**Walking of the cows related to the placement of the shafts**	**Not relevant at pasture**
	**Light and air quality of the lying area**	**Not relevant at pasture**
	**Position of animals in the cubicles (diagonal and lying, backward-forward standing, diagonal and standing, backward-forward and standing).**	**Not relevant at pasture**
	**Comfortable, safe and clean flooring.**	**Not relevant at pasture**
	**Run (after release from restraint.)**	**Not relevant at pasture**
	**Stumble, fall**	**Not relevant at pasture**
	**Building allowing mounting**	**Not relevant at pasture**
	**Access to outdoor loafing area**	**Not relevant at the pasture**
	**Lameness**	**Accepted**
	**Broken tail**	**Accepted**
	**Blind eye**	**Accepted**
	**Ingrown horn**	**Accepted**
	**Abrasions, injuries, integument alteration (hairless patches, lesions/swellings) **	**Accepted**
	**Coughing**	**Accepted**
	**Nasal discharge**	**Accepted**
	**Diarrhoea**	**Accepted**
	**Ocular discharge**	**Accepted**
	**Claw conformation**	**Limited practical application**
	**Skin irritation**	**Limited practical application**
	**Signs of Facial eczema**	**Limited practical application**
	**Thick hocks **	**Not relevant at pasture**
	**Thick carpi**	**Not relevant at pasture**
	**Animals needing further care**	**Not relevant at pasture**
	**Lying down and standing difficulty**	**Not relevant at pasture**
	**Abnormal sitting position (dog sitting)**	**Not relevant at pasture**
	**Abomasal dislocations**	**Not relevant at pasture**
	**Bloated rumen**	**Not relevant at pasture**
**Behaviour**	**Social agonistic behaviour (headbutting, displacement, chasing, chasing up, fighting, pushing)**	**Accepted**
	**Fear behaviour towards human approach/Avoidance distance**	**Accepted**
	**Lapping behaviour/allogrooming**	**Accepted**
	**Milking behaviour (cow restlessness during milking; number kicking and stepping over a complete milking time)**	**Accepted**
	**Orientation in the collecting yard (facing towards the parlour, facing directly away from the parlour, not orientated towards the parlour,)**	**Accepted**
	**Qualitative behaviour (Active, Frustrated, Irritable, Relaxed, Friendly, Uneasy, Fearful, Bored, Sociable, Agitated, Playful, Apathetic, Calm, positively occupied, Happy, Content, Lively, Distressed, Indifferent, Inquisitive)**	**Accepted**
	**Animal handling during milking (vocal tone, hitting, tail pulling, etc.)**	**Accepted**
	**Leg stretching while standing in the pasture**	**Limited practical application**
	**Tail hanging straight and relaxed in pasture**	**Limited practical application**
	**Avoidance distance at the feeding rack**	**Limited practical application**
	**Stockperson to animal ratio**	**Limited practical application**

## Appendix B

Record-based assessments identified as potentially useful as part of an on-farm welfare assessment protocol from six welfare assessment protocols, four studies of on-farm welfare assessment protocols and the New Zealand Dairy Cattle Code of Welfare.

(1) What is the distance to the farthest paddock? ………………………….
(2) Do you mix the cows between the herds? Yes…….. No…….
(3) Do you use pain relief during routinely husbandry procedures? Yes…… No…….
(4) How do you manage your vaccination record?
(5) What do you use for cattle handling (e.g., prod, sticks, miscatch, tail twist, etc)?
(6) Is your stockperson trained in animal handling, professionalism and knowledge of signs and symptoms of diseases?
(7) How often do you cull your cows (planned/unplanned culls per year, enforced culls, e.g., TB (Tuberculosis))?

*Yearly Records*

(1) Number of mastitis/year
(2) Number of lameness/year
(3) Total mortality per year
(4) Milk somatic cell count
(5) Downer cow
(6) Milk fever
(7) Acetonemia
(8) Abortions
(9) Mortality at birth
(10) Retention of placenta
(11) Prolapse
(12) Infertility
(13) Dystocia

## Appendix C

Measurements included in the test protocol with method of assessment (and domain of coverage [6]).

### Appendix C.1. Nutrition

**Body Condition Scoring:** All animals will be scored using the Dairy NZ body condition score scale from 1 to 10 [32,43] (modified for final protocol; Table 2).

**Rumen Fill Score:** All animals will be scored using the Dairy Veterinary Consultancy Ltd. Rumen Fill Scorecard which scores rumen fill on a 1–5 scale [44] (modified for final protocol; Table 2).

**Average distance to water points:** Two paddocks near where the cows were being kept and which had two water troughs were examined and the average distance between the two paired troughs was calculated.

**Cleanliness of the trough:** (paddocks/feed pads): Visual observation in the paddocks and feed pads (if present). *Clean*: Transparent water, base easily visible, no apparent dirt/dead insects. *Marginal:* Base of the trough partly obscured, no floating dirt or dead insects. *Dirty*: Base of the trough not visible, dirt/insects may be observed. Note: Fresh green grass in trough does not affect category.

### Appendix C.2. Environment

**Cleanliness of the cows:** For all cows, dirtiness around flanks (including tail), lower and upper hind leg, udder and tails were scored using the four-point Wisconsin hygiene scoring system [27] (modified for final protocol; Table 2).

**Noise level inside milking parlour**: 0: No noise from the machinery/dogs. 1: Slight noise, conversation with milkers can be heard with concentration. 2: High noise, conversation cannot be heard.

**Availability of shelter**: A shelterbelt was defined as an area of trees where the size of the horizontal canopy radius was at least 12 times the height of the trees in the area. For farms with at least one group of trees which satisfied this condition (from a visual estimate), the main orientation of the shelterbelts was recorded using a compass.

The vertical canopy radius was estimated as per [45] for two representative areas of shelterbelt, and the protection provided by the shelterbelt was recorded separately for shelterbelts running north-south and east-west using three categories: 0, complete protection, 1, some protection, 2, no protection.

In addition, where individual trees had been planted for shade in more than 80% of paddocks, two representative paddocks were selected and the height (as per [45]) and the average horizontal radius (by pacing) of four trees per paddock were measured.

**Maximum waiting time before entering the milking parlour**: The start and end of the milking time will be assessed by the assessor inside the parlour (modified for final protocol, see Table 2).

**Environment assessments removed during the second phase of the study**

**Slope before entry and exit of the parlour**: Slope of the junction between the yard (both entry and exit) and the track will be assessed.

**Heat Stress indicators**: Symptoms like open mouth panting, laboured breathing, sweating and restlessness will be assessed.

**Cold stress symptoms**: Symptoms like huddling, shivering and facing away from wind or rain will be assessed.

Environment assessments added during the second phase of the study

**Space allowance in the collecting yard:** Yard area calculated from measurements of length and width (rectangular yards) and radius (circular yards). Space allowance was yard area/cow. Maximum herd size (from questionnaire).

**Proper use of the backing gate:** Speed of the backing gate measured at the end of milking, with the time taken for the backing gate to move a known distance (from one identifiable point to another (e.g., pair of railings) being measured.

**Head position of the cows in the collecting yard:** Proportion of cows standing in the collecting yard with their heads up, recorded immediately after one movement of the backing gate.

**Track condition**: Track surface condition, the slope of camber and the width of the track will be assessed as per [46]. All tracks within 100 m of the parlour will be assessed at ~20 m intervals. The score is the average for the width and camber and for surface condition the worst area is assessed.

### Appendix C.3. Health

**Lameness**: All cows will be locomotion scored using the 1–4 DairyNZ locomotion score [43].

**Broken tail**: Visual assessment for broken tails (swelling and or deviation) while cows are standing to be milked.

**Skin injuries:** All cows will be observed during milking for abrasions, cuts, hairless patches and swellings and the proportion of cows with any such injury recorded (modified for final protocol, see Table 2).

**Coughing**: Number of cows coughing during milking will be recorded.

**Blind eye**: Number of cows with blind eye will be recorded (modified for final protocol, see Table 2).

**Ingrown horn**: Number of cows with ingrown horns will be recorded (modified for final protocol, see Table 2).

**Health assessments removed during the second phase of the study**

**Nasal Discharge:** Proportion of cows with visible discharge from the nose will be recorded.

**Ocular discharge:** Proportion of cows with visible discharge from the eyes will be recorded.

**Diarrhoea**: Proportion of cows with faecal stains below the tail head on both sides of the tail will be assessed.

### Appendix C.4. Behaviour

**Social agonistic and affiliative behaviour in the paddocks:** Cows will be observed for a total of 30 min in the paddock prior to milking. The number of observed behaviours will be recorded. Social agonistic behaviour: headbutting, chasing, displacement, fighting, pushing. Affiliative behaviour: Lapping behaviour/allogrooming.

**Avoidance distance**: Average distance between the assessor and the point of aversion by the cow will be recorded [11] (modified for final protocol, see Table 2).

**Handling of cows during milking:** Vocal tone and aggressive behaviour by farm staff towards the cows will be assessed. The number of behaviours such as shouting, threatening and hitting the cows will be recorded during milking.

**Handling of the cows on track:** Use of dogs or quad bikes to move the cows forcefully will be observed from behind the stockperson. Movement will be considered forceful if the quad bikes or dogs are used to pressure the cows on the tracks. Pressure will be identified when the cows being pressed lift their heads.

**Behavioural assessments removed during the second phase of the study**

**Milking behaviour:** Cow restlessness during milking; number kicking and stepping over a complete milking time will be recorded.

**Orientation of the cows:** Orientation of the cows in the collecting yard (facing towards the parlour, facing directly away from the parlour, not orientated towards the parlour) will be recorded at the beginning of milking.
